# A glimpse on *Mycoplasma* species circulating in wild and captive bird communities in Egypt: prevalence and phylogenetic analyses

**DOI:** 10.1007/s11259-025-10844-3

**Published:** 2025-08-13

**Authors:** Rasha Abotaleb, Sherif Marouf, Dina Y. H. ELShafey, Nayera M. Al-Atfeehy, Hassan Aboul-Ella, Heidy Abo Elyazeed

**Affiliations:** 1 Department of Microbiology, Faculty of Veterinary Medicine, Cairo University, Giza, 12211 Egypt; 2 Animal Health Research Institute, Giza, 12618 Egypt

**Keywords:** Wild birds, Captive birds, *Mycoplasma gallisepticum*

## Abstract

Wild and captive birds are well known as possible carriers of numerous pathogens, and they have recently received scientific attention concerning human health. *Mycoplasma* spp. Infections have been detected in a variety of wild and captive bird species worldwide. The present work aimed to study the prevalence of *Mycoplasma* spp. in wild and captive birds in Egypt; a total of 250 birds were examined, representing 15 species (11 orders) of wild birds. The collected samples include 100 tracheal swabs from wild birds and 150 from captive birds. Mycoplasmas were detected using microbiological culture methods. PCR amplification of the 16S rRNA gene was performed for genus-specific identification of mycoplasmas, using universal primers for genus *Mycoplasma*. Selected PCR products were sequenced for phylogenetic analysis. *M. gallisepticum* and *M. synoviae* were also detected by specific PCRs targeting the 16S rRNA gene. Regarding the obtained result, 38/250 positive samples were obtained for the isolation of *Mycoplasma* spp., with a prevalence of 15.2%. Moreover, confirmation with PCR tests using GPO primers and DNA extracted from the isolates resulted in 22/250 (8.8%) positive samples, which came from birds that belonged to six orders, where the highest occurrence was in Galliformes, followed by Strigiformes, Columbiformes, Accipitriformes, Bucerotiformes, and Ciconiiformes, respectively. Four tested birds were positive for *Mycoplasma gallisepticum* (*M. gallisepticum*), and one was positive for *Mycoplasma synoviae* (*M. synoviae*). *M. gallisepticum* and *M. synoviae* were only detected in two orders (Accipitriformes and Galliformes). The phylogenetic analysis revealed a high variability of *Mycoplasma* spp. and strong similarities between the studied Egyptian strains and other reference strains available in GenBank.

**Supplementary Information:**

The online version contains supplementary material available at 10.1007/s11259-025-10844-3.

## Introduction

The public health interest in birds as pathogen vectors and their role in disease epidemiology has increased due to new infections in wildlife and their possible zoonotic hazard (Evans et al. 2005; Benskin et al. 2009). Mycoplasmas are the smallest self-replicating organisms found widely in nature. There are more than 100 species in the genus *Mycoplasma*; most of them are commensals or opportunistic pathogens, although some seem more pathogenic than others, relevant to the severity of the associated disease, and hence their effect on production and their economic consequences, as well as the frequency of occurrence worldwide (Swayne et al. 2013; Pereyre and Tardy 2021). *Mycoplasma* spp. cause various clinical symptoms, including pulmonary, reproductive, conjunctivitis, arthritis, and skeletal problems (Luttrell and Fischer 2007; Noel et al. 2010). *Mycoplasma* spp. can cause detrimental diseases in chickens and result in significant financial losses for the global poultry industry. *Mycoplasma* spp. are considered non-pathogenic or a component of the upper respiratory tract microbiota in other bird species, such as white storks, raptors, and several kinds of waterfowl. These species of birds frequently exhibit these microbes in healthy individuals.

Some wild bird species may have respiratory pathogens such as *M. gallisepticum* in house finches, even though healthy individuals of these species do not have these organisms (Fischer et al. 2022). *M. gallisepticum* and *M. synoviae*, two well-known poultry mycoplasmas that are thought to be the most pathogenic, have also been found in various wild bird species and represent different orders (Kursa et al. 2024). Since many mycoplasmas are host-specific, infections caused by *M. gallisepticum* and *M. synoviae* primarily cause clinical signs in the wild species of Galliformes (Luttrell et al. 1992, Bradbury et al. 2001a, b; Welchman et al. 2002, 2013; Bencina et al. 2003; Forrester et al. 2011; Cookson and Shivaprasad 2016). The house finch (*Haemorhous mexicanus*) population in the USA experienced epidemic conjunctivitis in 1994 because of *M. gallisepticum*, which had a high fatality rate and a high prevalence. Up to 60% of the house finch population perished before the illness stabilized at a low prevalence level (Hochachka et al. 2021).

Wild waterfowl are considered carriers and reservoirs of numerous bacteria and viruses that can cause different diseases. A single pathogen can typically infect a wide variety of bird species belonging to many orders. Nonetheless, it is well-recognized that *Mycoplasma* spp. are host-specific microbes. Bird hosts are typically *Mycoplasma* spp. vectors; however, the infection does not cause illness symptoms in them (Gharaibeh and Hailat 2011). The transmission of mycoplasmosis between domestic and wild birds in the same order has been documented in many previous reports (Sawicka et al. 2020; Sawicka-Durkalec et al. 2022). Only wild turkeys showed clinical indications of mycoplasmosis (Davidson et al. 1982), such as respiratory problems and sinusitis, which was diagnosed as *M. gallisepticum*. Later, it was documented more frequently in game birds of the order Galliformes, including pheasants (Welchman et al. 2002; Bencina et al. 2003; Cookson and Shivaprasad 2016). *Mycoplasma* spp. were isolated from 82% of nestlings, 26% of juveniles, and 50% of adults in a study of injured or debilitated birds of prey in Germany. Using an immunobinding assay (IBA), these species were identified as *M. gypis*, *M. meleagridis*, *M. falconis*, and *M. buteonis*; five isolates were unidentifiable (Lierz et al. 2000).

Mycoplasmas were found in birds from the Rio de Janeiro Zoo that did not exhibit any respiratory symptoms, and the frequency of *M. synoviae* was higher than that of *M. gallisepticum*. Among the orders examined, there were differences in *Mycoplasma* spp., *M. gallisepticum*, and *M. synoviae*, with the highest prevalence in prey-seeking birds, followed by Galliformes and Piciformes. *Mycoplasma* spp. found in Rio de Janeiro Zoo’s avian population attests to the agents’ circulation and the necessity of additional research on *Mycoplasma* spp. dissemination in zoos for *Mycoplasma* spp. epidemiological analysis (Magalhães et al. 2020). Molecular approaches have been utilized in conjunction with bacteriological isolation, as these bacteria are laborious, slow to grow exponentially, and require a richer culture medium. PCR does not require isolation from clinical specimens and is quick, sensitive, and inexpensive to run in the laboratory (Islam et al. 2011; Umar et al. 2017).

The objectives and the highlights of the current study are as follows: (I) To represent a pioneer contribution to wildlife, more specifically, wild birds, a study in Egypt was conducted, as there is very limited knowledge in this scientific field in Egypt. Also, there are a clear rareness in studies that have been performed on wild and captive birds in Egypt and our study represents one of the headmost studies to investigate the prevalence of *Mycoplasma* spp. in wild birds in Egypt; (II) to survey a wide variety of both wild and captive birds belonging to different orders, families, and species for the occurrence of *Mycoplasma* spp. from the wild birds’ focal areas in Egypt; (III) to elucidate the actual situation of the presence of *M. gallisepticum* and *M. synoviae* in wild and captive birds in Egypt as many mycoplasmas are known to be host-specific, infections brought on by *M. gallisepticum* and *M. synoviae* mostly elicit clinical symptoms in Galliformes wild species; and (IV) to analyze the genetic relatedness of selected *Mycoplasma* spp.—positive samples and to figure out their phylogeny to previously found strains from other parts of the world.

Beyond any doubt, the obtained results will be very important at both the national and international levels, as studies targeting neglected host species are always valuable for the development of public health strategies. This knowledge acts as a building block that equips the scientific community with a provisional view to be cautious of hidden threats that may be visible and disastrous one day through the carriers of zoonotic diseases, and/or host range spillover.

## Materials and methods

### The involved birds’ categorization

The current work procedures have been conducted from June 2022 to June 2024. A total of 250 wild and captive birds were involved in the current study; 100 of them were wild birds, and the other 150 were captive birds from the Giza Zoo. Regarding the sample size calculation in both wild and captive birds as it is not possible to know the size of the wild population, for a surveillance study, therefore it was sufficient to sample an indefinite number of birds based on availability as well as in the case of captive birds due to the limited captive population in Giza Zoo, all available captive birds were sampled. A sampling of both wild and captive birds was done in their eventual habitat, and samples were properly transported to the laboratory for further microbiological investigation. The wild group represented ten species and eight orders, while the captive group represented five species and three orders, as mentioned in Table 1.

**Table 1:** The total number of samples examined that were collected from different wild and captive birds, illustrating the order, species, and the common name of each involved bird

Type of Bird	Order	Species	Common Name	Number of Samples
Wild birds	Pelecaniiformes	*Bubulcus ibis*	Cattle egret	10
	Gruiformes	*Gallinula galeata*	Common gallinule	10
	Strigiformes	*Athene noctua*	Little owl	10
	Charadriiformes	*Hoplopterus spinosus*	Spur-winged lapwing	10
	Accipitriformes	*Elanus caeruleus*	Black-winged kite	10
	Charadriiformes	*Larus argentatus*	European herring gull	10
	Columbiformes	*Streptopelia risoria*	Barbary dove	10
	Bucerotiformes	*Upupa epops*	Eurasian hoopoe	10
	Coraciformes	*Merops orientalis*	Asian green bee-eater	10
	Charadriiformes	*Himantopus himantopus*	Black-winged stilt	10
Captive birds	Galliformes	*Pavo cristatus*	Indian peafowl	10
	Galliformes	*Gallus gallus domesticus*	Cochin chicken	20
	Galliformes	*Gallus gallus domesticus*	Silkie chicken	40
	Galliformes	*Gallus gallus domesticus*	European chicken	20
	Galliformes	*Numida meleagris*	Helmeted guineafowl	20
	Ciconiiformes	*Ciconia ciconia*	White stork	10
	Anseriiformes	*Alopochen aegyptiaca*	Egyptian goose	30
Total	11	15		250

### Collecting samples from the involved birds

A total of 250 tracheal swabs were collected from live birds, as the tracheal and choanal cleft swabs are the best sample in live birds for most *Mycoplasma* spp. All samples were tested as soon as possible after collection. In the case of transportation, when necessary, the collected swabs were vigorously agitated in 1–2 mL of *Mycoplasma* broth and then discarded. An ice pack or some other means of chilling was included as primary isolation of *M. gallisepticum* and *M. synoviae* can be affected at room temperature (Ball et al. 2020). 0.45 μm syringe filtration and serial dilutions of the samples were made in *Mycoplasma* broth because the presence of specific antibodies, antibiotics, or inhibitory substances in the tissues may inhibit *Mycoplasma* growth.

### Phenotypic identification of the obtained isolates

Broth and agar media were used for the transportation, primary isolation, and cultivation of *Mycoplasma* spp. from the collected samples. The following broth and agar media were used for transportation, primary isolation, and cultivation of *Mycoplasma* from the collected samples (OIE 2004; OIE 2008):

- Part A: Pleuropneumonia-like organism (PPLO) broth base in distilled water without crystal violet (21 g/L).
- Part B: 150 mL swine serum (inactivated by heating at 56 °C for 1 h), 100 mL (25% w/v) fresh yeast extract, 10 mL (10% w/v) glucose solution, 10 mL (5% w/v) thallium acetate, 5 mL (200,000 International Units (IU)/mL) penicillin G, and 20 mL (0.1% w/v) phenol red solution. The pH is adjusted to 7.8. A mix of 1 mL (10% v/v) NAD solution and 1 mL (10% v/v) cysteine solution is also added for primary isolation of *M. synoviae*. Part A was autoclaved at 121 °C, at 1 atmospheric pressure for 15 min, and, after cooling, was added to part B, which had previously been sterilized by filtration using a 0.25 μm Seitz filter. For the corresponding solid medium, 10 g of purified agar, known to support the growth of *Mycoplasma*, was added to part A above. The mixture was autoclaved as before and kept in a water bath at 56 °C. The constituents of part B, omitting the phenol red, were mixed separately and then incubated at 56 °C. Parts A and B were mixed carefully to avoid the production of air bubbles and were dispensed into 50 mm dishes using 7–9 mL/dish. Excess surface moisture was removed by a short incubation at 37 °C. Plates were stored in the refrigerator at approximately 4 °C for up to 4 weeks.

Specimens were inoculated onto both *Mycoplasma* agar via the running drop technique and were also inoculated in broth. The solid medium may help detect slow-growing *Mycoplasma* and *Mycoplasma*-like colonies, which can be overgrown by saprophytes in broth. Inoculated plates were incubated at 37 °C in a sealed gas pack jar. Increased humidity and CO_2_ concentration in the atmosphere have been reported to enhance growth, and these conditions were obtained via the inclusion of damp cotton wool as a source of humidity and by flushing the container with 5–10% CO_2_ in nitrogen by placing a lighted candle in the container. The caps of the liquid medium containers were tightly sealed before incubation to avoid spurious changes in pH. The cultures were kept for at least 20 days before discarding. Broth cultures were examined daily for turbidity and/or changes in color, as most mycoplasmas, including *M. gallisepticum* and *M. synoviae*, metabolize sugar-producing acids, causing a change in the pH of the medium from red/orange to yellow. Other mycoplasmas hydrolyze arginine, creating alkaline conditions and causing a change in pH, which consequently changes the broth color from red/orange to strong red or fuchsia. Any observable growth in the broth was subcultured onto a solid medium immediately. If there was no color change after 7–14 days, the broth was subcultured onto the solid medium. This was carried out because the presence of an arginine-hydrolyzing (alkali-producing) *Mycoplasma* spp. may mask the acid’s color change produced by *M. gallisepticum* or *M. synoviae*, or there might be *Mycoplasma* strains with a less active metabolic status. During prolonged cultivation on solid growth medium, some *Mycoplasma* species form web-like structures called “films and spots”, that was evaluated during the microscopic examination of the obtained cultures. Biochemical tests, including glucose fermentation, arginine hydrolysis, and digitonin sensitivity tests, were used to assist in the identification and differentiation of *Mycoplasma* spp. from other related species, but they are not specific for *M. gallisepticum* or *M. synoviae*.

### DNA extraction from the obtained isolates

All presumed positive samples with colonies resembling fried eggs underwent PCR testing as a confirmatory step. The DNA from samples with typical *Mycoplasma* colonies was extracted from the PPLO agar using the QIAamp DNA Mini kits (Qiagen, Hilden, Germany) by following the instructions provided by the manufacturer. Thereafter, the DNA was stored at − 20 °C for further molecular analysis.

### PCR for molecular confirmation

The reaction mixture was prepared according to the manufacturer’s instructions (Marouf et al. 2022). For each sample, the following solutions were dispensed into a PCR tube: 45 µL of mix containing 0.4 µM of each primer for 16S rRNA *M. gallisepticum* and *M. synoviae* or 0.2 µM for 16S rRNA GPO, as shown in Table 2. The appropriate extracted DNA sample (5 µL) was added to each tube. An extracted DNA from previously identified *Mycoplasma* species was used as a positive control, while nuclease-free water was used as a negative control. The tubes were then placed in a thermal cycler (ESCO life sciences, Singapore) for the following cycles: 40 cycles: 94 °C for 30 s, 55 °C for 30 s, and 72 °C for 60 s; 1 cycle (final extension): 72 °C for 5 min and hold at 4 °C. PCR amplification products were detected via conventional gel electrophoresis, incorporating appropriate size markers, and the 5 µg/ml ethidium bromide (EtBr, Sigma-Aldrich, UK)-stained products were visualized under UV light.

**Table 2:** The used primer sequences are associated with the expected amplicon size

Primers	Sequences	Expected Amplicon	References
**GPO**: *Mycoplasma* *group specific*	F: (5′-GGGAGCAAACAGGATTAGATACCCT-3′) R: (5′-TGCACCATCTGTCACTCTGTTAACCTC-3′)	280 bp	van Kuppeveld et al. 1992
**MG**: *Mycoplasma* *gallisepticum*	F: (5′-GAGCTAATCTGTAAAGTTGGTC-3′) R: (5′-GCTTCCTTGCGGTTAGCAAC-3′)	185 bp	OIE 2019
**MS**: *Mycoplasma* *synoviae*	F: (5′-GAGAAGCAAAATAGTGATATCA-3′) R: (5′-CAGTCGTCTCCGAAGTTAACAA-3′)	210 bp	OIE 2019

### Sequencing and phylogenetic analysis

The phylogenetic analysis was performed using representative samples selected from different species of wild birds and captive birds, based on the band quality. The selected PCR products were sent for sequencing as described by Ramírez et al. (2008), and each amplification product was sequenced with reverse amplification primers. A phylogenetic analysis was conducted using the neighbor-joining algorithm with the Kimura 2-parameter model based on amino acid sequence (Kimura 1980). The reliability of phylogenetic inference at each branch node was estimated via the bootstrap method with 1000 replications. An evolutionary analysis was conducted using MEGA6 (Tamura et al. 2013). The obtained *Mycoplasma* spp. sequences through the current work were deposited in the GenBank database under the following accession numbers: OR143719, OR143720, OR143721, OR143722, OR143723, OR143724, OR143725, and OR143726.

### Statistical analysis of the obtained data

Data were collected, entered, and tabulated using the Microsoft Excel software. Data were then imported into the Statistical Package for the Social Sciences (SPSS version 21.0) software for category prevalence, total prevalence, Chi-square, and statistical significance calculations.

## Results

### Phenotypic identification of the obtained isolates

The current study revealed 118/250 (47.2%) positive samples for *Mycoplasma*-like colonies isolated with typical fried egg colonies. Only three species (European herring gull, Asian green bee-eater, and black-winged stilt) were found to be negative via primary isolation. *Mycoplasma* spp. were distinguished from *Acholeplasma* spp. using the digitonin test; *Mycoplasma* isolates (digitonin sensitive) were 32.2% (38/118) and originated from ten species and seven orders (cattle egret, little owl, black-winged kite, Barbary dove, Eurasian hoopoe, Indian peafowl, Cochin chicken, Silkie chicken, Helmeted guineafowl, and white stork). A total of 38 *Mycoplasma* isolates were subjected to biochemical tests such as glucose fermentation, film and spot formation, and arginine hydrolysis. The obtained results revealed that most of the isolates were positive for glucose fermentation (35/38), while 18 isolates were positive for film and spot formation, and three isolates were only positive via the arginine hydrolysis test.

### PCR for molecular confirmation

By using 16S rRNA gene (GPO) primers in a PCR assay, 22 out of 38 DNA samples were confirmed as *Mycoplasma* spp. (57.9%). The positivity for *Mycoplasma* spp. was different among the studied orders, where the highest occurrence was in Galliformes (Indian peafowl, Cochin chicken, Silkie chicken, and Helmeted guineafowl) followed by Strigiformes (Little owl), Columbiformes (Barbary dove), Accipitriformes (black-winged kite), Bucerotiformes (Eurasian hoopoe), and Ciconiiformes (white stork), respectively. All the *Mycoplasma* spp. positive samples were tested for *M. gallisepticum* and *M. synoviae*, which are considered the major pathogens of commercial poultry. Although the overall prevalence of both species was quite low, there were higher prevalences of *M. gallisepticum* than *M. synoviae*, where only four samples were positive for *M. gallisepticum* (two from black-winged kite, one from Cochin chicken, and one from Indian peafowl), and one sample was positive for *M. synoviae*, which was detected in Indian peafowl, as shown in Table 3.

**Table 3:** Isolation and PCR detection of *Mycoplasma* spp., *M. gallisepticum*, and *M. synoviae* categorized according to the common names of wild and captive birds in Egypt

Type of Birds	Birds Tested for* Mycoplasma* Species	% of Primary Isolation	Digitonin Test	PCR		
			%of *Mycoplasma* Isolates			
				GPO	MG	MS
Wild birds (10 species) (*n* = 100)	Cattle Egret	5/10 (50%)	2/5 (40%)	0/2	Not done	Not done
	Common Gallinule	3/10 (30%)	0/3 (0%)	Not done	Not done	Not done
	Little Owl	10/10 (100%)	4/10 (40%)	3/4	0/3	0/3
	Spur Winged lapwing	5/10 (50%)	0/5 (0%)	Not done	Not done	Not done
	Black Winged kite	5/10 (50%)	2/5 (40%)	2/2	2/2	0/2
	European Herring gull	0/10 (0%)	0/0 (0%)	Not done	Not done	Not done
	Barbary Dove	7/10 (70%)	3/7 (42.9%)	3/3	0/3	0/3
	Eurasian Hoopoe	4/10 (40%)	1/4 (25%)	1/1	0/1	0/1
	Asian green bee-eater	0/10 (0%)	0/0 (0%)	Not done	Not done	Not done
	Black winged stilt	0/10 (0%)	0/0 (0%)	Not done	Not done	Not done
Subtotal	10	39/100 (39%)	12/39 (30.7%)	9/12 (75%)	2/9 (22,0.2%)	0/9 (0%)
Captive birds (5 species) (*n* = 150)	Indian peafowl	4/10 (40%)	2/4 (50%)	2/2	1/2	1/2
	Cochin chicken	14/20 (70%)	5/14 (35.7%)	3/5	1/3	0/3
	Silkie chicken	18/40 (45%)	6/18 (33.3%)	3/6	0/3	0/3
	European chicken	7/20 (35%)	5/7 (71.4%)	0/5	Not done	Not done
	Helmeted guineafowl	8/20 (40%)	7/8 (87.5%)	4/7	0/4	0/4
	White stork	8/10 (80%)	1/8 (12.5%)	1/1	0/1	0/1
	Egyptian goose	20/30 (66.6%)	0/20 (0%)	Not done	Not done	Not done
Subtotal	5	79/150 (52.6%)	26/79 (32.9%)	13/26 (50%)	2/13 (15.4%)	1/13 (7.7%)
Total	15 species	118/250 (47.2%)	38/118 (32.2%)	22/38 (57.9%)	4/22 (18.1%)	1/22 (4.5%)

The overall prevalence of GPO, MG, and MS, as detected by PCR, was as follows: 22/250 (8.8%) were positive for GPO, 4/250 (1.6%) were positive for MG, and 1/250 (0.4%) were positive for MS. Also, 118/250 (47.2%) were positive for cultural isolation in both wild and captive birds as an overall prevalence. The total number of digitonin test-positive samples was 38/250 (15.2%).

### Sequencing and phylogenetic analysis

**Fig. 1:**
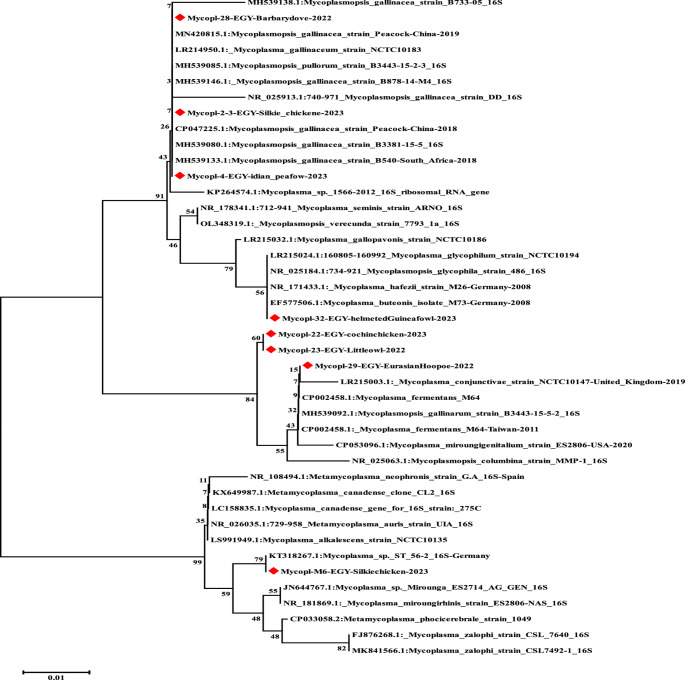
Phylogenetic tree of avian *Mycoplasma* strains in Egypt during 2022–2023 and reference isolates from the GenBank. A phylogenetic analysis was conducted on the 16S-23S rDNA intergenic spacer regions (ISRs) using the neighbor-joining algorithm with the Kimura 2-parameter model based on amino acid sequence. The reliability of phylogenetic inference at each branch node was estimated using the bootstrap method, which had 1000 replications. An evolutionary analysis was conducted using MEGA6 (http://www.megasoftware.net). Accessed on December 17/2023. A red rhomboid indicates isolates sequenced specifically for this study

A clean unsmeared single band PCR products on gel electrophoresis were selected to be sequenced to perform a phylogenetic analysis of eight selected sequences and to construct a phylogenetic tree as shown in Fig. 1; Table 4; the results revealed two main clusters with high similarity among the samples and selected strains in GenBank (91.1–100%) as shown in Supplementary Table 1. The first cluster was divided into two sub-clusters; the first sub-cluster contained four isolates (one from a wild bird and three from captive birds); isolates from Barbary dove, Silkie chicken, Indian pea-fowl (GenBank accession numbers: OR143719, OR143725, and OR143726) were closely related, 100% similarity to *Mycoplasma gallinaceum* detected in peacocks from China and South Africa in 2018 (GenBank accession numbers: MN420815, CP047225, and MH539133) and *Mycoplasma pullorum* isolated in South Africa in 2018 (GenBank accession numbers: MH539085.1). In addition, one isolate from Helmeted guinea fowl (GenBank accession number: OR143723), which has a 99% similarity to *Mycoplasma buteonis* and *Mycoplasma hafezii* that were detected in Germany in 2008 (GenBank accession numbers: EF577506 and NR_171433) and *Mycoplasma glycophila* that was detected in the USA in 2008 (GenBank accession numbers: NR_025184.1). While the second sub-cluster contained three isolates (two from wild birds and one from a captive bird), the isolates were taken from little owl, Eurasian hoopoe, and Cochin chicken (GenBank accession numbers: OR143722, OR143720, and OR143721), which have high similarities to each other and were closely related, 99.5% similarity, to *Mycoplasma fermentans* detected in Taiwan in 2011 (GenBank accession number: CP002458) and *Mycoplasma gallinarum* detected in South Africa in 2018 (GenBank accession number: MH539092.1). The second main cluster contained one isolate from Silkie chicken (GenBank accession number: OR143724), which showed 100% similarity to a German isolate (GenBank accession number: KT318267). Additionally, strong sequence identity was also observed in the same isolate and an undescribed *Mycoplasma* sp. Mirounga (GenBank accession number: JN644767) is shown in Table 5.

**Table 4:** Tabulation of the obtained phylogenetic data, including the reference number, date, country, species, the obtained isolate, and the isolation source

Reference number	Date	Country	Species	The obtained isolate	Isolation source
MN420815	08/9/2019	China	Peacock	*Mycoplasma gallinaceum*	Trachea
CP047225	31/12/2019	China	Peacock	*Mycoplasma gallinaceum*	Trachea
MH539133	27/6/2018	South Africa	-	*Mycoplasma gallinaceum*	-
EF577506 and NR_171433	24/4/2007, 17/4/2008	Germany	Peregrine falcon (Falco peregrinus)	*Mycoplasma buteonis and Mycoplasma hafezii*	Trachea
CP002458	27/12/2010	Taiwan	A non-AIDS patient with acute respiratory disease	*Mycoplasma fermentans*	Respiratory discharges
CP002458	27/12/2010	Taiwan	A non-AIDS patient with acute respiratory disease	*Mycoplasma fermentans*	Respiratory discharges
CP002458	27/12/2010	Taiwan	A non-AIDS patient with acute respiratory disease	*Mycoplasma fermentans*	Respiratory discharges
KT318267	20/7/2015	Germany	White stork (Ciconic Ciconia)	*Mycoplasma* sp.	Trachea

**Table 5:** Summarization, tabulation, and highlights of the obtained sequencing data

Sample number	The obtained sequence size	GenBank accession number of the obtained sequence	GenBank accession number of the compared sequence	Sequence identity	The identity strain	The obtained isolate
1	192 bp	OR143719	MN420815	100%	Peacock20181011	*Mycoplasma gallinaceum*
2	192 bp	OR143725	CP047225	100%	Peacock20181011	*Mycoplasma gallinaceum*
3	192 bp	OR143726	MH539133	100%	B540-15-5-3	*Mycoplasma gallinaceum*
4	190 bp	OR143723	EF577506 and NR_171433	99%	M73, M26	*Mycoplasma buteonis* and *Mycoplasma hafezii*
5	192 bp	OR143722	CP002458	99.5%	M64	*Mycoplasma fermentans*
6	192 bp	OR143720	CP002458	99.5%	M64	*Mycoplasma fermentans*
7	192 bp	OR143721	CP002458	99.5%	M64	*Mycoplasma fermentans*
8	192 bp	OR143724	KT318267	100%	ST 56 − 2	*Mycoplasma* spp.

## Discussion

Wild birds are considered significant hosts of *Mycoplasma* spp., though limited data are available compared to birds of commercial interest. *Mycoplasma* spp. have been identified as one of the emerging bacterial pathogens affecting migratory birds (Parin et al. 2019). The current work represents the first study highlighting *Mycoplasma* spp.‘s prevalence in wild and captive birds in Egypt. The current work’s findings showed 38/250 positive samples for the isolation of *Mycoplasma* spp., with a prevalence of 15.2% using conventional PCR to confirm *Mycoplasma* spp. detected in 22/250 positive samples with a prevalence of 8.8% as shown in Table 3. From all *Mycoplasma* spp. positive samples recorded in the current study, only four out of 22 isolates were positive for *M. gallisepticum*, with a prevalence of 18.1%, which were isolated from Black-winged kite, Cochin chicken, and Indian peafowl.

While *M. synoviae* was identified only in one isolate (4.5%) from the Indian peafowl, as shown in Table 3, the obtained results disagree with Sawicka-Durkalec et al. (2021), who aimed to determine the occurrence of *Mycoplasma* spp. in wild birds, and they recorded that 498 positive samples for *Mycoplasma* spp. were found to be negative for *M. gallisepticum* or *M. synoviae*, while the current study corroborates with the results of Taiyari et al. (2023), who aimed to screen for the occurrence of *M. gallisepticum* and *M. synoviae* among various species of birds in Malaysia and found that 3/54 (5.6%) samples were positive for *M. gallisepticum*, detected using culture and PCR, and *M. synoviae* was detected in 16/56 (29.6%) and 22/54 (40.7%) samples via culture and PCR, respectively. Moreover, Magalhães et al. (2020) investigated *Mycoplasma* spp. in 81 birds at the Rio Zoo from different orders. They found that all birds were negative for isolation, while 51/81 birds were positive for *Mycoplasma* spp. using PCR with a prevalence of 62.96%. In this study, the prevalence of *M. synoviae* was higher than *that of M. gallisepticum*, which contrasts with the results obtained in the current work, where the percentage of *M. gallisepticum* was found to be higher than that of *M. synoviae*. It is worth mentioning that isolation of *M. synoviae* is more difficult than *M. gallisepticum*, which could explain the higher isolation rate of *M. gallisepticum* in the current work. If the tracheal swabs had undergone direct DNA extraction and been tested via *M. synoviae* and *M. gallisepticum* PCR, it is possible that *M. synoviae* detection rates could have been higher in the current study as well.

The difference between the obtained results and those reported by Magalhães et al. (2020) may be related to differences in bird species, method of diagnosis, and geographical location. The current study found that *Mycoplasma* spp. detected in a broad range of species which may play a critical role as a vector of disease and that supports the result of Sawicka-Durkalec et al. (2021) who provide information on wild bird species that could be considered potential reservoirs or carriers of *M. gallisepticum* and could be useful to determine the course of future studies on the phylogeny and spread of *M. gallisepticum* in various hosts. On the other hand, Ziegler et al. (2017), in a study performed on 97 birds, found that in other bird species, such as Common Nightingales (*Luscinia megarhynchos*) and tits (Paridae), *Mycoplasma* spp. are absent in healthy birds.

In the current work, for the molecular diagnoses of *M. gallisepticum* by PCR technique, two pairs of PCR primers were used as shown in Table 2 and consisted of a universal primer pair for the genus *Mycoplasma* depending on 16S rRNA sequences and the second pair was specific for *M. gallisepticum* according to (Aghabalaei et al. 2012; Malekhoseini et al. 2017; Mahmmoud et al. 2022), and the efficacy of these primers was confirmed by (Hamad et al. 2019a, b; Al-dabbagh et al. 2021), but the primers used for the detection of *M. gallisepticum* could amplify other closely related species. Ideally, targets other than 16S rRNA should be used prospectively to overcome this limitation and confirm the detection of *M. gallisepticum*. However, the usual *M. gallisepticum* DNA sequence targets are 16S rRNA (Lauerman 1998), defined as a reference target (OIE 2019); other target sequences have been used, such as the *mgc*2 gene (García et al. 2005). The latter was defined as a more specific target, in contrast with PCR primers for the highly conserved 16S rRNA sequence that might amplify other *M. gallisepticum*-like species hosted by birds, such as *Mycoplasma imitans* (García et al. 2005), *Mycoplasma tullyi* (Yavari et al. 2017), and other unidentified species detected in seabirds (Ramírez et al., 2019).

The prevalence of isolates confirmed by PCR from *Mycoplasm*a spp. in correspondence with culture is 57.9% which contradicts the results obtained by Muhammad et al. (2018) that showed higher detection sensitivity towards the PCR and correspond to the results obtained by Hamzah et al. (2022) that showed nearly a similar proportion of the prevalence by PCR and culture, 63%. It is well-established that DNA extraction for *Mycoplasma* molecular identification can be performed directly from samples or broth culture or agar culture. The sensitivity and specificity obtained from DNA extraction from agar cultures are the highest in comparison with the DNA extraction from both the samples themselves and the broth cultures. Therefore, the current study was based on DNA extraction from agar cultures. The only obstacle that would adversely affect the sensitivity of the GPO in the current work is the difficulties associated with DNA extraction from agar, as the extraction of DNA from the microscopic *Mycoplasma* colonies cultured on agar plates is difficult because the plasticity of *Mycoplasma* enables agar penetration. This eventually causes cell loss during the harvesting of colonies from the agar surface, leading to an overall loss of the obtained biomass, which, by its role, affects the DNA concentration, which, by its role, affects the PCR results and their correspondence to culture results (Sakmanoglu et al. 2017).

Although the current work results revealed that the highest occurrence of *Mycoplasma* spp. were in Galliformes (Indian peafowl, Cochin chicken, Silkie chicken, and Helmeted guineafowl) followed by Strigiformes (Little owl), Columbiformes (Barbary dove), Accipitriformes (Black-winged kite), Bucerotiformes (Eurasian hoopoe), and Cicoiniiformes (White stork), respectively, no statistical significance between those groups was obtained by Chi-square statistical analysis at *p* <.05. These results are similar to findings reported by (Magalhães et al. 2020; Sawicka-Durkalec et al. 2021), which showed a higher occurrence of other *Mycoplasma* spp. in some orders of birds, such as Charadriiformes, Ciconiiformes, Accipitriformes, and Falconiformes, than in Passeriformes and others. In the current study, two *Mycoplasma* strains were isolated from Cattle egrets via culture, which corresponds to the results of Le Gall-Ladevèze et al. (2021), who detected *Mycoplasma* spp. DNA from Cattle egrets. *Mycoplasma* spp. were not detected in common gallinule and spar-winged lapwing via isolation, while all fried egg colonies were classified as *Acholeplasma* spp.

Regarding the order Strigiformes (little owl), *Mycoplasma* spp. were detected using isolation and PCR techniques (4/10 and 3/10), respectively, but the species were not identified; this result agrees with the result obtained by Magalhães et al. (2020), who recorded 25% of *Mycoplasma* spp. from the order Strigiformes. On the contrary, Sawicka-Durkalec et al. (2021) did not detect any *Mycoplasma* spp. from owls.

Charadriiformes, especially the Laridae family, are known as carriers of many viral and bacterial pathogens. However, only limited reports describe the occurrence of *Mycoplasma* spp. in gulls (Kempf et al. 2000); in the conducted investigation, *Mycoplasma* spp. were not detected in Charadriiformes (Spar-winged lapwing, black-winged stilt, and European herring gull), which contrasts with Sawicka-Durkalec et al. (2021). While Ramírez et al. (2023) isolated six *Mycoplasma* strains from tracheal swabs taken from four different species of sea birds. Four strains originated from three yellow-legged gulls (*Larus michahellis*) and Cory’s shearwater (*Calonectris borealis*) from Spain, one strain from a South African Kelp gull (*Larus dominicanus*), and one strain from an Italian black-headed gull (*Chroicocephalus ridibundus*). *Mycoplasma* spp. have been detected in Accipitriformes (Black-winged kite); this result is in good agreement with Magalhães et al. (2020) and Sawicka-Durkalec et al. (2021). From *Columbiformes* (Barbary Dove), three strains of *Mycoplasma* spp. were isolated and confirmed via PCR, and similar results were obtained in racing pigeons (Sawicka et al. 2019).

Also, *Mycoplasma* spp. have been detected in Eurasian hoopoes, but no strains were recovered from Asian green bee-eaters. *Mycoplasma* spp. could be isolated from captive birds, where most isolates were in Galliformes (two isolates were recovered from Indian peafowl, three from Cochin chicken, three from Silkie chicken, and four from Helmeted guinea fowl). *Mycoplasma* spp. were also detected in white stork, which is congruent with the results reported by Möller et al. (2016). On the other side, Fischer et al. (2022) found that *Mycoplasma* spp. are considered non-pathogenic or a component of the upper respiratory tract microbiota in white storks. Concerning European chicken and Egyptian goose, no *Mycoplasma* spp. were isolated. Meanwhile, Prüter et al. (2018) did not detect any *Mycoplasma* spp. in the pharyngeal swabs of the Egyptian goose (*Alopochen aegyptiacus*).

The phylogenetic analysis and phylogenetic tree of eight selected *Mycoplasma* strains are illustrated in Fig. 1, and the results revealed two main clusters with high similarity among samples and selected strains in GenBank (91.1–100%) as shown in Supplementary Table 1. It should be noted that this analysis is based only on one gene, and a more detailed phylogeny using whole genome sequencing (WGS) might provide higher discriminatory power. The results also revealed a high variability of *Mycoplasma* spp. The occurrence of *Mycoplasma* spp. in a broad range of wild and captive birds, with higher prevalence in captive birds (52,6%) than wild birds (39%), verifies the circulation of these agents and the necessity for more research on *Mycoplasma* distribution in wild birds in wildlife and captive birds in zoos for epidemiological analysis. The results demonstrate that wild and captive birds are important vectors of numerous *Mycoplasma* spp. pathogenic to domestic poultry. Furthermore, wild and captive birds could transfer non-pathogenic microbes, which may have significant consequences, as reported by Trinh et al. (2018). This *Mycoplasma* sp. is host-specific for sea elephants despite its presence in an isolate from Silkie chicken, which may be related to the workers’ movement in the zoo. This means that wild and captive birds act as vectors and reservoirs or may be commensals to *Mycoplasma* spp.

## Conclusions

According to the results obtained, it is concluded that the estimated prevalence of *Mycoplasma* spp. in wild birds is different between wild and captive birds, and it depends on the method of detection and the species of bird. Also, the phylogenetic analysis revealed a high variability of *Mycoplasma* spp. and a high similarity to different isolates from different locations around the world, which sheds light on the role of migratory, wild, and captive birds in mycoplasmosis epidemiology. Also, the obtained results point fingers at wild and captive birds in Egypt, which could play a certain role in spreading diseases in the environment. After all, further studies are needed to establish the link between infections in domestic, wild, and captive birds. Due to the scarce contribution of the Egyptian scientific community in the field of wildlife and more specifically, the wild birds, the current contribution through the conducted study and the obtained findings could be considered a leading work in this field, which, by its role, fills a clear scientific gap and prospectively could be a cornerstone for further contributions in the same field.

## Electronic supplementary material

Below is the link to the electronic supplementary material.

Supplementary Material 1

## Data Availability

All generated and analyzed data were presented clearly in the submitted manuscript. Sequence data that support the findings of this study have been deposited in the GenBank with the primary accession codes: OR143719, OR143725, OR143726, OR143723, OR143722, OR143720, OR143721, and OR143724.
