# Supplementary material for: A glimpse on Mycoplasma species circulating in wild and captive bird communities in Egypt: prevalence and phylogenetic analyses
Source: Vet Res Commun. 2025 Aug 13;49(5):280. doi: 10.1007/s11259-025-10844-3 (PMC12350449; doi:10.1007/s11259-025-10844-3)
Supplement: Supplementary file 1 — Supplementary Material 1 [file 11259_2025_10844_MOESM1_ESM.docx]

| **Identity Percent** | | | | | | | | | | | | | | | | | | |  |
| --- | --- | --- | --- | --- | --- | --- | --- | --- | --- | --- | --- | --- | --- | --- | --- | --- | --- | --- | --- |
|  | **1** | **2** | **3** | **4** | **5** | **6** | **7** | **8** | **9** | **10** | **11** | **12** | **13** | **14** | **15** | **16** | **17** |  |  |
| **1** |  | 96.4 | 95.8 | 93.2 | 95.8 | 100.0 | 100.0 | 95.8 | 95.8 | 96.9 | 100.0 | 95.8 | 96.9 | 96.9 | 93.2 | 100.0 | 100.0 | **1** | **M-gallinacea-Peacock-China-2018** |
| **2** | 3.7 |  | 99.5 | 91.7 | 99.5 | 96.4 | 96.4 | 92.2 | 92.2 | 93.2 | 96.4 | 99.5 | 99.5 | 99.5 | 91.7 | 96.4 | 96.4 | **2** | **M-fermentans-M64-Taiwan-2011** |
| **3** | 4.3 | 0.5 |  | 92.2 | 99.0 | 95.8 | 95.8 | 91.7 | 91.7 | 92.7 | 95.8 | 99.0 | 99.0 | 99.0 | 92.2 | 95.8 | 95.8 | **3** | **M-miroungigenitalium-ES2806-USA-2020** |
| **4** | 7.2 | 9.1 | 8.4 |  | 91.1 | 93.2 | 93.2 | 90.1 | 90.1 | 91.1 | 93.2 | 92.2 | 91.1 | 91.1 | 100.0 | 93.2 | 93.2 | **4** | **Mycoplasma sp. ST 56-2-Germany-2015** |
| **5** | 4.3 | 0.5 | 1.0 | 9.6 |  | 95.8 | 95.8 | 91.7 | 91.7 | 92.7 | 95.8 | 99.0 | 99.0 | 99.0 | 91.1 | 95.8 | 95.8 | **5** | **M-conjunctivae-NCTC10147-UK-2019** |
| **6** | 0.0 | 3.7 | 4.3 | 7.2 | 4.3 |  | 100.0 | 95.8 | 95.8 | 96.9 | 100.0 | 95.8 | 96.9 | 96.9 | 93.2 | 100.0 | 100.0 | **6** | **M-gallinacea-Peacock-China-2019** |
| **7** | 0.0 | 3.7 | 4.3 | 7.2 | 4.3 | 0.0 |  | 95.8 | 95.8 | 96.9 | 100.0 | 95.8 | 96.9 | 96.9 | 93.2 | 100.0 | 100.0 | **7** | **M-gallinacea-B540-SouthAfrica-2018** |
| **8** | 2.2 | 6.1 | 6.7 | 8.5 | 6.7 | 2.2 | 2.2 |  | 100.0 | 99.0 | 95.8 | 91.7 | 92.7 | 92.7 | 90.1 | 95.8 | 95.8 | **8** | **M-hafezii-M26-Germany-2008** |
| **9** | 2.2 | 6.1 | 6.7 | 8.5 | 6.7 | 2.2 | 2.2 | 0.0 |  | 99.0 | 95.8 | 91.7 | 92.7 | 92.7 | 90.1 | 95.8 | 95.8 | **9** | **M-buteonis-M73-Germany-2008** |
| **10** | 2.1 | 6.0 | 6.6 | 8.4 | 6.6 | 2.1 | 2.1 | 0.0 | 0.0 |  | 96.9 | 92.7 | 93.8 | 93.8 | 91.1 | 96.9 | 96.9 | **10** | **Mycopl-32-EGY-helmetedGuineafowl-2023** |
| **11** | 0.0 | 3.7 | 4.3 | 7.2 | 4.3 | 0.0 | 0.0 | 2.2 | 2.2 | 2.1 |  | 95.8 | 96.9 | 96.9 | 93.2 | 100.0 | 100.0 | **11** | **Mycopl-28-EGY-Barbarydove-2022** |
| **12** | 4.3 | 0.5 | 1.1 | 8.4 | 1.0 | 4.3 | 4.3 | 6.7 | 6.7 | 6.6 | 4.3 |  | 99.0 | 99.0 | 92.2 | 95.8 | 95.8 | **12** | **Mycopl-29-EGY-EurasianHoopoe-2022** |
| **13** | 3.2 | 0.5 | 1.0 | 9.6 | 1.0 | 3.2 | 3.2 | 5.5 | 5.5 | 5.5 | 3.2 | 1.0 |  | 100.0 | 91.1 | 96.9 | 96.9 | **13** | **Mycopl-22-EGY-cochinchicken-2023** |
| **14** | 3.2 | 0.5 | 1.0 | 9.6 | 1.0 | 3.2 | 3.2 | 5.5 | 5.5 | 5.5 | 3.2 | 1.0 | 0.0 |  | 91.1 | 96.9 | 96.9 | **14** | **Mycopl-23-EGY-Littleowl-2022** |
| **15** | 7.2 | 9.1 | 8.4 | 0.0 | 9.6 | 7.2 | 7.2 | 8.5 | 8.5 | 8.4 | 7.2 | 8.4 | 9.6 | 9.6 |  | 93.2 | 93.2 | **15** | **Mycopl-M6-EGY-Silkiechicken-2023** |
| **16** | 0.0 | 3.7 | 4.3 | 7.2 | 4.3 | 0.0 | 0.0 | 2.2 | 2.2 | 2.1 | 0.0 | 4.3 | 3.2 | 3.2 | 7.2 |  | 100.0 | **16** | **Mycopl-2-3-EGY-Silkiechickene** |
| **17** | 0.0 | 3.7 | 4.3 | 7.2 | 4.3 | 0.0 | 0.0 | 2.2 | 2.2 | 2.1 | 0.0 | 4.3 | 3.2 | 3.2 | 7.2 | 0.0 |  | **17** | **Mycopl-4-EGY-idianpeafow-2023** |
|  | **1** | **2** | **3** | **4** | **5** | **6** | **7** | **8** | **9** | **10** | **11** | **12** | **13** | **14** | **15** | **16** | **17** |  |  |

**Supplementary Table 1.** Nucleotide sequence identities (percentage) of partial 16S rRNA gene sequences of the selected *Mycoplasma* isolates compared to other selected strains available in GenBank.
